# Antiviral Drug Discovery To Address the COVID-19 Pandemic

**DOI:** 10.1128/mBio.02134-20

**Published:** 2020-09-25

**Authors:** Douglas D. Richman

**Affiliations:** a University of California San Diego, Distinguished Professor of Pathology and Medicine (Active Emeritus), Director, The HIV Institute, Co-Director, San Diego Center for AIDS Research, Florence Seeley Riford Chair in AIDS Research (Emeritus), La Jolla, California, USA; Washington University School of Medicine

**Keywords:** coronavirus, COVID-19, SARS-CoV-2, antiviral agents, drug resistance, hepatitis C virus, human immunodeficiency virus, protease inhibitors

## Abstract

The magnitude of the morbidity and mortality inflicted upon the global population in less than 1 year has driven the inescapable conclusion that the discovery and development of effective antiviral drugs for COVID-19 are urgent and should be prioritized. The antiviral drug discovery programs that emerged for HIV and hepatitis C virus have enabled technology and expertise to accelerate this process for SARS-CoV-2. The description of candidate lead inhibitors for the viral main protease (M^pro^) exemplifies this accelerated approach and reminds us of the needs and opportunities for addressing this pandemic.

## INTRODUCTION

The need for more effective antiviral drugs against SARS-CoV-2 is undeniable. Even with the identification of the first effective drug for coronavirus infection, remdesivir, the need for additional drugs remains critical. Regardless of whether or when a vaccine becomes available, antivirals for SARS-CoV-2 will still be needed for several reasons: the unlikelihood that a vaccine will be 100% effective, the incompleteness of vaccine coverage because of both vaccine hesitancy and the numerous logistical challenges to accomplishing prompt large-scale immunization of the majority of the population, the possibility of limited durability of vaccine protection, the need for additional prophylaxis for high-risk subjects and poor vaccine responders, and the future value of effective antiviral treatment for Middle East respiratory syndrome (MERS) and new coronaviruses that will likely emerge from zoonoses. Unmet needs for new antivirals include additional and greater efficacy, oral bioavailability, utility for prophylaxis as well as treatment, and the knowledge that combination therapy can enhance efficacy and prevent the emergence of drug resistance. Although drugs to modulate the late immunopathological complications are also needed, only highly active antiviral drugs will be beneficial for prophylaxis, the treatment of early disease, and perhaps the reduction of transmissibility.

Remdesivir targets the viral RNA-dependent RNA polymerase (RdRp), and additional inhibitors of that enzyme are in development, as mentioned below. As viral enzymes are critical for replication and represent the majority of targets of all approved antiviral drugs, other coronavirus enzymes represent obvious candidate targets for effective drugs. With protease inhibitors proven as effective for antivirals against HIV and hepatitis C virus (HCV), the two coronavirus proteases, the main protease (M^pro^) and the papain-like protease (PL^pro^), also stand out as clear candidate targets for antiviral drug discovery.

Hattori et al. (1) examined two indole-chloropyridinyl-ester derivatives, GRL-0820 and GRL-0920, which they had designed against SARS-CoV before the emergence of SARS-CoV-2. The M^pro^s of the two viruses share significant sequence and structural similarities. To their credit, Hattori et al. use multiple assays to document activity (inhibition of infectivity, replication, and cytopathic effects) in two different cell lines, and importantly, they conscientiously characterize cellular toxicity. Too many publications fail to carefully recognize that the reduction of replication that is being measured may be attributable to diminished host cell viability. In fact, the data in the work of Hattori et al. suggest that the purported activity against SARS-CoV-2 of the two HIV protease inhibitors, lopinavir and nelfinavir, is probably attributable to cellular toxicity. Moreover, the claims of activity for chloroquine and hydroxychloroquine may be attributable to cellular toxicity according to the data in the work of Hattori et al. They also confirm with structural modeling and high-performance liquid chromatography and mass spectrometry (HPLC/MS) that their inhibitors of M^pro^ dock and then covalently bind in the catalytic site. This information will provide invaluable guidance for the modification of the inhibitors when designing yet more potent derivatives. In addition to the identification of compounds by Hattori et al. (1), Dai et al. have described inhibitors of M^pro^ with nanomolar activity against SARS-CoV-2 (2).

Of note, Hattori et al. (1) found no significant anti-SARS-CoV-2 activity for several compounds reportedly active against SARS-CoV-2, including lopinavir, nelfinavir, nitazoxanide, favipiravir, and hydroxychloroquine, while the proven drug remdesivir displayed activity at nontoxic concentrations. It has been frustrating to observe the widespread rush to use these drugs both for patient management and in numerous redundant clinical trials, when the *in vitro* data should be sufficient to discourage this enthusiasm, especially when their *in vitro* potency against coronaviruses is so low compared to their potency against their proven targets.

The urgency of curbing the COVID-19 pandemic has motivated a large number of investigators globally to identify new drugs and repurpose old ones to target viral proteins, as well as some host cell targets essential for viral replication. The only antiviral yet shown to have clinical efficacy is the RdRp inhibitor remdesivir. These studies were conducted in subjects with moderate to severe disease, while efficacy with antiviral drugs generally is greatest when administered early in disease. The results of planned studies with outpatient administration to subjects at high risk for severe disease, of aerosolized administration, and of remdesivir in combination with interferon beta-1a are eagerly awaited. Other ribonucleoside inhibitors of RdRp, which are orally bioavailable, unlike remdesivir, have entered clinical trials. MK-4482 (formerly EIDD-2801) has activity against SARS-CoV, MERS, and SARS-CoV-2 in mice and is currently being studied in humans (3). MK-4482 appears to act by inducing frequent nucleoside transitions during RNA replication, resulting in increased error rates, which probably account for impaired replication. AT-527 is a prodrug of a guanosine ribonucleotide analog, which was well tolerated with substantial activity in studies of HCV-infected subjects (4). It has *in vitro* activity against SARS-CoV-2, justifying its investigation for COVID-19 (5).

Early studies with parenteral interferon beta-1b (6) and inhaled interferon beta (as yet unpublished) showed encouraging results. Numerous clinical trials are being conducted with specific antibodies, both from convalescent-phase plasma and as monoclonal antibody preparations, but no randomized controlled trials to document activity clinically have yet been published. Repurposing already available drugs is appealing because the availability of the drug along with pharmacokinetic and toxicity profiles facilitates expeditious evaluation for COVID-19. As mentioned above, several such drugs have been and remain under study because of ready availability rather than compelling *in vitro* activity against SARS-CoV-2. An exception is camostat mesylate, which has been approved for the treatment of pancreatic cancer in Japan. Camostat mesylate inhibits the host serine protease TMPRSS2, which dramatically enhances SARS-CoV-2 replication by proteolytically cleaving the spike (S) protein of the virus to activate the spike to mediate cell entry (7). With human safety and pharmacokinetics already characterized, camostat mesylate is in several clinical trials as a repurposed drug for COVID-19.

The severe complications of COVID-19 appear to be more the consequence of a misguided, pathological host immune response to ongoing viral infection (8). Numerous studies are in progress to identify effective modulators of this dysregulated immune response with some early indications of efficacy of the corticosteroid dexamethasone (9). The potential benefits of inhibitors of interleukin-6 and other cytokines and chemokines are being examined in multiple clinical trials, but evidence of clinical efficacy is pending.

The Mitsuya group has a track record of developing drugs that are effective against HIV infection. These include the reverse transcriptase inhibitors ddC (zalcitabine) and ddI (didanosine), the protease inhibitor darunavir, and the reverse transcriptase translocation inhibitor islatavir (10–12). With the need for antiviral drugs for COVID-19, we can all hope that not only will this group be successful in identifying effective coronavirus drugs, but that numerous other investigators are successful in finding drugs against multiple targets. We have learned with other viral diseases that to address the challenges of safety, efficacy, and drug resistance, to tackle the demands of both prophylaxis and treatment, and to manage both the viral replication and immunopathological aspects of COVID-19, numerous drugs will be needed. Let us all applaud the many efforts and wish many successes in the battle against this pandemic.
